# Resurgence of influenza and respiratory syncytial virus in Egypt following two years of decline during the COVID-19 pandemic: outpatient clinic survey of infants and children, October 2022

**DOI:** 10.1186/s12889-023-15880-9

**Published:** 2023-06-05

**Authors:** Amr Kandeel, Manal Fahim, Ola Deghedy, Wael H. Roshdy, Mohamed K. Khalifa, Rabeh El Shesheny, Ahmed Kandeil, Amel Naguib, Salma Afifi, Amira Mohsen, Khaled Abdelghaffar

**Affiliations:** 1 Ministry of Health and Population, Cairo, Egypt; 2 Central Public Health Laboratory, Ministry of Health and Population, Cairo, Egypt; 3 Centre of Scientific Excellence for Influenza Viruses, National Research Centre, 12622 Dokki, Giza, Egypt; 4 Consultant Ministry of Health and Population, Cairo, Egypt; 5 National Research Center, Cairo, Egypt

**Keywords:** Influenza viruses, Respiratory Syncytial virus, Acute respiratory infection, Outbreak, Survey

## Abstract

**Introduction:**

Two years after unprecedented low rates of circulation of most common respiratory viruses (SARS-CoV-2), the Egyptian ARI surveillance system detected an increase in acute respiratory infections (ARIs) with a reduced circulation of the severe acute respiratory syndrome coronavirus 2 (SARS-CoV-2), especially among school children. A national survey was conducted to estimate the burden and identify the viral causes of ARIs among children < 16 years of age.

**Methods:**

A one-day survey was carried out in 98 governmental outpatient clinics distributed all over Egypt 26 governorates. The four largest referral hospitals in each governorate where most influenza-like illness (ILI) patients seek care were selected. Using the WHO case definition, the first five patients < 16 years of age with ILI symptoms visiting the selected outpatient clinics on the survey day were enrolled. Basic demographic and clinical data of patients were collected using a linelist. Patients were swabbed and tested for SARS-CoV-2, influenza, and Respiratory Syncytial virus (RSV) by RT-PCR at the Central Laboratory in Cairo.

**Results:**

Overall, 530 patients enrolled, their mean age was 5.8 ± 4.2, 57.1% were males, and 70.2% reside in rural or semi-rural areas. Of all patients, 134 (25.3%) had influenza, 111 (20.9%) RSV, and 14 (2.8%) coinfections. Influenza-positive children were older compared to RSV, (7.2 ± 4.1, 4.3 ± 4.1, p < 0.001), with more than half of them (53.0%) being school students. Dyspnea was reported in RSV more than in influenza (62.2% vs. 49.3%, p < 0.05). Among RSV patients, children < 2 years had a higher rate of dyspnea than others (86.7% vs. 53.1%, < 0.001).

**Conclusions:**

A resurgence of influenza and RSV was detected in Egypt in the 2022–2023 winter season. Influenza caused a higher rate of infection than RSV, while RSV caused more severe symptoms than influenza. Monitoring a broader range of respiratory pathogens is recommended to estimate the ARI burden and risky groups for severe disease in Egypt.

## Introduction

The COVID-19 pandemic has been associated with changes in the natural course of non-SARS-CoV-2 respiratory infections depending on the virus type worldwide. The reduction in non-SARS-CoV-2 acute respiratory infections (ARI), especially influenza and respiratory syncytial virus (RSV), reported during the first two years of the COVID-19 pandemic was related to the implementation of nonpharmaceutical interventions (NPI) [1]. However, early reports suggest that ARI caused by non-SARS-CoV-2 respiratory viruses has returned to normal in 2022–2023 as COVID-19 rates have declined [2] .

Children are commonly affected by ARI which is one of the important causes of pediatric death [3]. Viruses cause more than 80% of ARI in infants and young children and are the second leading cause of infant mortality worldwide [4]. Influenza and respiratory syncytial virus (RSV) are common viruses that can lead to pediatric respiratory tract infections and cause local epidemics, especially in schools, and nurseries [5]. In spite of being less severe, non-hospitalized influenza is associated with frequent use of antipyretics, antibiotics, and injections, as well as increased child absenteeism and healthcare visits, resulting in a significant economic losses and burdens on healthcare systems [6–7].

Respiratory Syncytial Virus (RSV) is the leading cause of lower respiratory tract infections (LRTIs) in young children worldwide. It is also a major cause of pediatric infectious disease mortality in children younger than five with up to 200,000 deaths worldwide every year [8].

Influenza vaccines offer highly effective protection and can help induce herd immunity to reduce transmission in populations. Seasonal influenza vaccination is recommended for everyone six months old and older as a preventative measure against serious influenza-related complications [9]. The Ministry of Health and Population (MoHP) of Egypt recommends influenza vaccination, particularly for pregnant women, the elderly, young children, and people with weak immune systems, who are most at risk of complications from influenza. MoHP assured that obtaining the annual influenza vaccine this year provides protection against 4 types of influenza viruses expected to spread this seasos [10].

Pharmaceutical companies are racing to obtain the United States Food and Drug Administration (FDA) approval for an RSV vaccine that can be administered to adults over 60 years old. The vaccine may be available as early as May 2023, and it will not be long before an RSV vaccine is available for all ages, according to experts [11].

MoHP is monitoring influenza and SARS-CoV-2 activities through ARI integrated surveillance, National infectious Diseases surveillance (NEDSS), ARI syndromic surveillance, ARI mortality surveillance, and event-based surveillance (EBS) systems [12]. Furthermore, in 2016, RSV was introduced to the sentinel surveillance to monitor RSV among hospitalized children under the age of 2 years [13].

Egypt sentinel surveillance reported higher viral infection rates (36%) during the first two years of the pandemic among ARI patients in outpatients and inpatients compared to the pre-pandemic period (17%), with the predominant virus being SARS-CoV-2 (31%), while influenza and RSV rates were unprecedentedly low (4% and 15% respectively) [13–15].

In October 2022, Egypt EBS detected high rates of school absenteeism with ARI symptoms among primary and preparatory school children in many Egyptian governorates. In addition, the ARI surveillance detected an increase in the circulation of influenza and RSV among the outpatients and inpatients (unpublished data). In late November 2022 MoHP reported an increase in the rate of RSV from 15% in 2020–2021 to 70% [15]. During a press conference, H.E. the Minister of Health and Population reported 1,611 RSV infections, mainly among young children [16]. Therefore, MoHP has conducted a one-day national survey in October 2022 to identify the viral causes of ARIs in Egypt among children < 16 years and estimate the burden of each circulating viral cause to help control measures and prevent future epidemics.

Study objectives were to describe the epidemiology and clinical profile of ARIs caused by influenza and RSV, estimate their burden, and identify changes in epidemiology associated with their resurgence after two years of low activity.

## Methods

### Study setting

The survey was conducted on the 14th of October 2022 in all 26 Egyptian governorates. The four largest referral hospitals outpatient clinics in each governorate where most of the influenza-like illness (ILI) patients seek healthcare were selected including infectious diseases hospitals, chest hospitals, and general and central hospitals.

### Study population

Each selected hospital surveillance team was requested to identify the first five patients < 16 years of age with ILI symptoms visiting the clinic on the survey day. Patients with ILI were identified by the World Health Organization case definition of fever > 38 °C and cough within 10 days of onset [17]. Patients whom their parents/guardians consented to participate in the survey were interviewed using a standard line list that include patient age, gender, governorate and district of residence, clinical symptoms, onset date, and history of comorbidities.

### Sample size calculation

Based on a 15% estimated prevalence of influenza in Egypt 2020 − 202 [13], a sample of at least 481 children < 16 years of age was required to provide a representative sample of this age group with a Confidence limit of ± 3% and a design effect of 2. The sample size was calculated using Epi info7 software. A study conducted in a Lower Egyptian district in 2013 showed that most ILI patients sought healthcare, so the study is supposed to provide a representative sample of the Egypt population < 16 years of age [18].

### Laboratory methods

Patients were asked to provide Nasopharyngeal and Oropharyngeal swabs (NP/OP) swabs in order to detect and treat acute respiratory viral infections as soon as possible. Specimens were kept in nitrogen tanks and stored in viral transport media (VTM) before being transported to Cairo’s Central Public Health Laboratory (CPHL) within 24 h for Real-Time polymerase chain reaction testing for RSV, influenza, and SARS-CoV-2 (RT-PCR).

Samples were collected using flocked nasopharyngeal/oropharyngeal swabs, which were then immersed in a viral transport medium before being delivered to CPHL for testing. In a nutshell, RNA was extracted from clinical samples using chemagic 360 technology (PerkinElmer Inc). SARS-CoV-2 RNA (N1 and N2 gene) was identified using a Viasure SARS-CoV-2 RT-PCR Detection Kit (Certest Biotec SL, Spain), followed by a Viasure respiratory viral panel I RT-PCR Detection Kit (Flu A, Flu B, and RSV) (Certest Biotec SL, Spain). According to CDC guidelines, positive influenza A samples were further investigated for influenza A RT-PCR subtyping [19–20].

### Statistical analysis

Categorical data were expressed in frequencies and proportions and continuous data were expressed as means and standard deviations. The proportion of influenza, RSV, coinfection, and negatively tested patients was calculated as the number of positive patients for each type divided by the number tested. Characteristics of different viral causes were compared to better describe the difference in the demographic, clinical picture, and severity. The statistical differences were tested using the chi^2^ test for qualitative variables and t-test and ANOVA for quantitative variables. All tests were two-sided, and P < 0.05 was considered statistically significant. All statistical analyses were performed using SPSS software (version 25.0).

## Results

Overall, 530 patients < 16 years of age with ILI symptoms enrolled on the survey day. Their mean age was 5.8 ± 4.2, 56.8% were 5–15 years of age, 57.4% were males, 209 (39.4%) were school students and 3.0% had a history of comorbidities. Of all patients, 260 (49.1%) were positive for one or more of the three tested viruses including 25.3% influenza viruses, 20.9% RSV, 2.6% RSV/influenza coinfection, and only one patient was positive for both SARS-CoV-2 and influenza A/H1N1 (Tables 1 and 2). By influenza subtype, 15.3% were influenza A/H3N2, 9.6% were A/H1N1, and two cases caused by influenza B (Table 1).

**Table 1:** Viral causes of acute respiratory infections among the Egyptian children < 16 years of age attending governmental Egyptian outpatient clinics, October 2022

Category	Number	Percent
**Total tested**	530	100.0
**Number positive**	260	49.1
**Influenza viruses**	134	25.3
** A/H3N2 subtype**	81	15.3
** A/H1N1 subtype**	51	9.6
**B subtype**	2	0.4
**Respiratory Syncytial Virus**	111	20.9
**Co-infection**	15	2.8

**Fig. 1:**
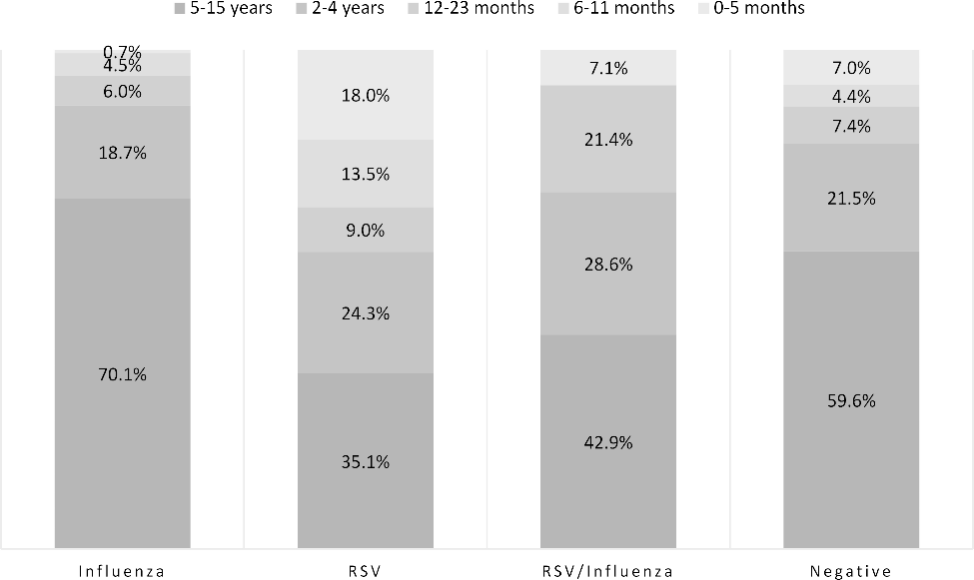
Age distribution of acute respiratory infections by viral causes among study participants

A comparison between influenza-positive, RSV-positive, children coinfected with RSV and influenza viruses (RSV/Influenza coinfection) and those who were negative for all tested respiratory viruses revealed that most of the influenza-positive and negatively tested children were ≥ 5 years of age (70.1% and 59.6%, respectively). While the majority of RSV-positive and coinfected patients were under the age of 5 years (64.9% and 57.1%, respectively), with 40.5% of RSV patients under 2 years old (Fig. 1).

Influenza-positive children were older compared to RSV, coinfected, and negatively tested (mean ages = 7.2, 4.3, and 6.1, p < 0.001), with more than half of influenza-positives (53.0%) being school students compared to 18.0% of RSV-positive, 28.6% of coinfected and 41.9% of negatively tested children (p < 0.001) (Table 2). Male sex predominated in all infections except for RSV, the difference in sex distribution was significant between influenza and RSV-positive children (male: female ratio = 1.7:1.0, p = 0.01).

Most of the patients (70.2%) were residents of rural or semi-urban areas in Upper and Lower Egypt governorates rather than the urban or frontier governorates. This is true for influenza-positive (69.5%), RSV-positive (66.6%), coinfected (64.3%), and negatively tested children (72.2%), with no difference, observed between different groups in the residence or percent with comorbidity (Table 2).

**Table 2:** Comparison between the demographic and epidemiologic characteristics of acute respiratory infections by viral causes among study participants

Characteristic	Total patients (n = 530)		Influenza (n = 134)		RSV (n = 111)		Influenza/RSV coinfection (n = 14)		Negative for influenza and RSV (n = 270)		P value
	Number	Percent	Number	Percent	Number	Percent	Number	Percent	Number	Percent	
**Age groups (years)**											
0–5 months	41	7.7	1	0.7	20	18.0	1	7.1	19	7.0	< 0.001
6–11 months	41	7.7	6	4.5	15	13.5	0	0.0	20	7.4	
12–23 months	114	21.5	8	6.0	10	9.0	3	21.4	58	21.5	
2–4 years	301	56.8	25	18.7	27	24.3	4	28.6	161	59.6	
5–15 years	33	6.3	94	70.1	39	35.1	6	42.9	12	4.4	
**Gender**											
Male	304	42.6	85	63.4	55	49.5	8	57.1	155	57.4	0.188
Female	226	57.4	49	36.6	56	50.5	6	42.9	115	42.6	
**Region**											
Upper Egypt	184	34.7	53	39.6	41	36.9	4	28.6	85	31.5	0.494
Lower Egypt	188	35.5	40	29.9	33	29.7	5	35.7	110	40.7	
Frontier	78	14.7	23	17.2	20	18.0	2	14.3	33	12.2	
Urban governorates	80	15.1	18	13.4	17	15.3	3	21.4	42	15.6	
**Comorbidity**	16	3.0	1	0.7	2	1.8	1	7.1	12	4.4	0.147
**Attending school or nursery**	209	39.4	71	53.0	20	18.0	4	28.6	113	41.9	< 0.001
**Symptoms**											
Fever	480	90.6	123	91.8	101	91.0	14	100.0	241	89.3	0.670
Cough	238	44.9	65	48.5	53	47.7	4	28.6	115	42.6	0.356
Sore throat	374	70.6	95	70.9	70	63.1	10	71.4	199	73.7	0.407
Rhinorrhea	284	53.6	59	44.0	60	54.1	10	71.4	155	57.4	0.048
Headache	10	1.9	3	2.2	2	1.8	0	0.0	5	1.9	0.984
Dyspnea	270	50.9	66	49.3	69	62.2	8	57.1	127	47.0	0.022
Vomiting	30	5.7	14	10.4	4	3.6	1	7.1	11	4.1	0.080
Diarrhea	21	4.0	3	2.2	6	5.4	0	0.0	12	4.4	0.600
Arthralgia	97	18.3	24	17.9	19	17.1	3	21.4	51	19.0	0.954
**Mean duration Onset-outpatient clinic visit**	4.0 ± 3.8		3.4 ± 2.6		3.9 ± 3.0		2.9 ± 1.4		4.4 ± 4.6		0.04

*RSV = Respiratory Syncytial virus

**Fig. 2:**
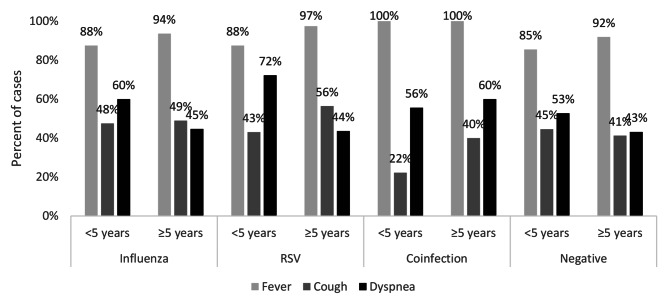
Clinical symptoms of acute respiratory infections by viral causes by age group among study participants

Clinically, more patients with RSV complained of rhinorrhea than influenza (54.1% vs. 44.0%, p = 0.06), while influenza patients presented more with vomiting compared to RSV patients (10.4% vs. 3.6%, p = 0.013). Dyspnea was reported in RSV more than in influenza, coinfection, and negatively tested patients (62.2% vs. 49.3%, 57.1%, and 47.0% respectively, p < 0.05). Among RSV, young children < 5 years and < 2 years had a higher rate of dyspnea than corresponding groups (72.2% and 86.7% vs. 43.6% and 53.1%, p = 0.01 and < 0.001 respectively) (Table 2; Fig. 2).

There were no significant differences between the four groups in terms of other clinical symptoms including fever, cough, sore throat, headache, nausea, diarrhea, and arthralgia (Table 2). The negatively tested patients took the longest time before seeking medical advice (mean duration 4.4 days), compared to influenza-positive and RSV-positive children who sought medical advice earlier with no significant difference between the two groups (3.4 vs. 3.9, p = 0.131) (Table 2).

## Discussion

We report a significant increase in the rates of influenza and RSV transmission in Egypt among children < 16 years who attended outpatient clinics after two years of interrupted transmission during the COVID-19 pandemic. We report a significant proportion of children > 2 years with RSV infection who missed their first exposure. RSV symptoms were more severe than influenza or coinfection especially among infants and young children.

A dramatic reduction in the spread of common respiratory viruses -including Influenza and RSV- was noted during the COVID-19 pandemic across different geographic regions and climate zones [21–26]. During the winter season, 2022–2023 different Northern Hemisphere countries have seen an increase in influenza and RSV infections, with different incidences, hospitalization rates, and circulation patterns due to differences in community preventive measures and immunity levels [27–28]. In the United States, RSV and influenza hospitalization rates surged 1.8 times higher than the highest cumulative in-season hospitalization rate for week 1 during previous seasons going back to 2010–2011 [29–30]. In Europe where influenza and RSV are currently prevalent, the World Health Organization warns that an influenza season epidemic is underway this winter earlier than expected, while COVID-19 remains a threat and this is expected to have a high impact on health services and populations. This study showed a significant increase in the rates of influenza and RSV among children < 16 years with ARI symptoms in Egypt outpatient clinics compared to the last two seasons and the pre-pandemic phase rates [13–14].

NPI such as staying at home and limiting social interaction was suggested to reduce respiratory viral spread [1]. Since both influenza and RSV are typically transmitted through close contact in closed childcare facilities and schools, school closures were thought to have contributed to the decline in influenza and RSV distribution among pediatric populations during the pandemic, and that lifting these restrictions has led to viruses resurging [2]. In accordance with this, our study found that attending school was associated with influenza infection. However, lifting COVID-19 restrictions cannot fully explain influenza and RSV reductions and resurgences, as other respiratory viruses, such as rhinoviruses and respiratory enteroviruses, were not affected by COVID-19. In addition, despite varying NPI strategies, influenza virus activity continued to be low throughout the pandemic period [31].

It is well-known that influenza viruses have the ability to continue circulating despite accumulated population immunity from infections and variable vaccination rates [2]. The sudden drop in influenza transmission during the COVID-19 pandemic was suggested to result in an evolutionary bottleneck affecting influenza’s genetic diversity where influenza resurgence has been associated with an increase in evolutionary pressure and genetic diversity at varying levels [2]. The viral competition, viral interference, and cross-immunity theories between respiratory viruses have also been proposed to explain influenza resurgence [32]. Additional studies are needed to better describe the reasons behind the dramatic changes in common respiratory viruses during and after the COVID-19 pandemic.

In agreement with other studies, we found that influenza transmission was higher among older than younger children, probably because the opportunity for onward spread is greater once children are in school [33–34]. The segmented influenza genome structure may also play a role in this, since segments can reassort with other circulating human and animal viruses. As a result, influenza viruses are able to undergo an antigenic shift, leading to new viruses that can easily reinfect people of all ages [35].

This study identified A/H3 as the predominated influenza subtype in the 2022–2023 influenza season. This pattern did not differ from what was reported in Egypt during the pre-pandemic phase [14]. Similar reports indicated the circulation of the same influenza subtypes in the Northern Hemisphere countries during the current winter season [36–37]. Virologic surveillance is necessary to monitor changes in influenza epidemiology including disease incidence and severity to help implement appropriate preventive strategies and provide valuable information about the yearly influenza vaccine composition.

It is believed that the resurgence of RSV was caused by the lack of exposure to RSV resulting in an immune naive cohort of RSV children under the age of five who used to be infected by age two. Whereas the waning of RSV immunity in older children and adults could make them more vulnerable to new infections. This could explain the resurgence of RSV reported in this study, raising concerns about increased healthcare demands [38].

Anyone can catch RSV however infants and children are particularly susceptible with 80% of children experiencing at least one RSV infection by the age of two [39]. This study reported a higher percentage of RSV among children < 5 years of age. This could be related to their low immunity due to lack of exposure during the last 3 years or the change in the epidemiology of the disease affected by the COVID-19 pandemic. The COVID-19 pandemic interrupted RSV transmission as discussed, and a partial interruption is expected after routine infant RSV vaccination is approved. In order to devise an effective preventive strategy including vaccination, dedicated RSV surveillance is needed to better understand RSV epidemiology and the risky group for severe disease to assist decision-makers in evaluating the impact of future vaccination [40]. This surveillance should include children up to the age of 15 so as to better describe the epidemiology including seasonality, age groups at risk, complications, and disease burden.

Studies indicated that RSV infection is a leading cause of serious illness among hospitalized children, causing greater morbidity and mortality, and has a more severe clinical course than influenza virus infection [41–43]. This study found that patients with RSV had a higher severity of disease as indicated by the higher frequency of dyspnea and the short duration before seeking medical advice. Broadening of Egypt ARI surveillance laboratory testing panel to include RSV and other respiratory viruses is recommended to better characterize the relative burden of ARI in Egypt. During RSV and influenza epidemics, it is particularly important to test for influenza and RSV to better identify the causative virus and prevent infection from spreading to elderly and chronically ill individuals [43–44].

Studies reported that children under 2 years are at a higher risk for severe and very severe RSV lower respiratory tract infections [45]. This study reported that children younger than 5 years of age and younger than 2 years of age were more likely to have dyspnea than those older. Children of younger ages should be considered a vulnerable population for future interventions, including future vaccination [45]. In addition, evaluation of the susceptibility of older children to severe RSV disease should be studied to better decide on the future vaccination strategy.

Available data suggest that among children, males are more likely to have severe seasonal influenza illness [46]. There was a higher male-to-female ratio among influenza-positive children with no differences found in RSV-positive children. However, no difference was found between males and females among influenza-positive children in the severity of symptoms indicated by dyspnea rates. Additional studies are required to investigate the role of sex differences in the severity of ARI among children and better identify risky groups.

As in previous studies, this study found no significant difference between patients infected with different causes of ARI regarding place of residence and clinical symptoms, except for influenza’s tendency to cause gastrointestinal symptoms [47–49].

On a clinical basis, attending physicians cannot distinguish between respiratory viruses causing respiratory tract infections, where differentiation is essential for avoiding inappropriate treatment and reducing viral transmission rate [43, 50]. Real-time genetic tests can differentiate respiratory viruses accurately as well as identify co-infection cases using highly sensitive real-time tests. Multiplex real-time quantitative reverse transcription PCR (qRT-PCR) is often included in combined COVID-19, influenza, and RSV testing kits. It is a highly sensitive and specific assay that uses multiple primers in the same reaction to amplify different nucleic acid sequences simultaneously, reducing costs, time, and resources [51].

Over the remaining COVID-19 pandemic period, it is unknown how respiratory viruses activity will change. With the lifting of travel restrictions, changes in NPI implementation policies, behavioral changes, waning population immunity, the emergence of new variants and subvariants of SARS-CoV-2, as well as the unavailability of influenza vaccine strains, it can be challenging to predict the future pattern of respiratory viruses activity [52]. The resurgence of influenza and RSV during the current winter season could predict a less intense next season since some of the immune deficiencies will have cleared. Nevertheless, it is not clear if COVID-19 will become a seasonal illness similar to influenza and RSV, or whether it will continue as it does now, with sporadic peaks throughout the year.

### Study limitations

The study is liable to at least four limitations. First, the survey was conducted in a single day, so the results do not reflect seasonality of the causative viruses. A second limitation is that the number of patients enrolled in each outpatient clinic is fixed. This makes it difficult to estimate the prevalence of various viral diseases accurately. The selection of four hospitals per governorate prevents the identification of the place distribution of causative agents. Lastly, the survey was conducted in the outpatient clinic settings which does not include ARI severe cases.

## Conclusions

We report a resurgence of influenza and RSV in children aged under 16 years in Egypt in winter 2022–2023, after two years of decline. Higher rates of infection have been identified than pre-pandemic rates that might overwhelm the healthcare system in Egypt. RSV found to have a higher incidence and more severe symptoms than influenza, especially in young children. The influenza vaccine is recommended for children who are at high risk. ARI surveillance should be maintained and expanded to include a wider range of respiratory pathogens and age groups to better assess each viral cause, facilitate decision-making, and monitor the changes in epidemiology and risky groups for severe acute respiratory infections.

## Data Availability

The datasets generated and/or analyzed during the current study are not publicly available to protect study subjects’ privacy but are available from the corresponding author (OD) upon reasonable request.

## List of Abbreviations

RSV: Respiratory Syncytial Virus
SARI: Severe Acute Respiratory Illness
RT-PCR: Real-Time polymerase chain reaction
MoHP: Ministry of Health and Population
SARS-CoV-2: Severe acute respiratory coronavirus 2
ICU: Intensive care unit
NEDSS: National infectious Diseases Surveillance
CPHL: Central Public Health Laboratories
LRTIs: lower respiratory tract infections
